# Quantitative Optical Coherence Tomography Angiography (OCTA) Parameters in a Black Diabetic Population and Correlations with Systemic Diseases

**DOI:** 10.3390/cells10030551

**Published:** 2021-03-04

**Authors:** Lincoln T. Shaw, Saira Khanna, Lindsay Y. Chun, Rose C. Dimitroyannis, Sarah H. Rodriguez, Nathalie Massamba, Seenu M. Hariprasad, Dimitra Skondra

**Affiliations:** 1 Department of Ophthalmology and Visual Science, University of Chicago Medical Center, Chicago, IL 60637, USA; Lincoln.Shaw@uchospitals.edu (L.T.S.); Saira.Khanna@uchospitals.edu (S.K.); Lindsay.Chun@uchospitals.edu (L.Y.C.); rose.dimitroyannis@gmail.com (R.C.D.); srodriguez5@bsd.uchicago.edu (S.H.R.); nmassamba@bsd.uchicago.edu (N.M.); retina@uchicago.edu (S.M.H.); 2 University of Chicago, Chicago, IL 60637, USA; 3 J. Terry Ernest Ocular Imaging Center, University of Chicago Medical Center, Chicago, IL 60637, USA

**Keywords:** diabetes mellitus, retinopathy, microvascular, complication, optical coherence tomography, angiography, black, African-American, systemic disease, biomarker

## Abstract

This is a cross-sectional, prospective study of a population of black diabetic participants without diabetic retinopathy aimed to investigate optical coherence tomography angiography (OCTA) characteristics and correlations with systemic diseases in this population. These parameters could serve as novel biomarkers for microvascular complications; especially in black populations which are more vulnerable to diabetic microvascular complications. Linear mixed models were used to obtain OCTA mean values ± standard deviation and analyze statistical correlations to systemic diseases. Variables showing significance on univariate mixed model analysis were further analyzed with multivariate mixed models. 92 eyes of 52 black adult subjects were included. After multivariate analysis; signal strength intensity (SSI) and heart disease had statistical correlations to superficial capillary plexus vessel density in our population. SSI and smoking status had statistical correlations to deep capillary plexus vessel density in a univariate analysis that persisted in part of the imaging subset in a multivariate analysis. Hyperlipidemia; hypertension; smoking status and pack-years; diabetes duration; creatinine; glomerular filtration rate; total cholesterol; hemoglobin A1C; and albumin-to-creatinine ratio were not significantly associated with any OCTA measurement in multivariate analysis. Our findings suggest that OCTA measures may serve as valuable biomarkers to track systemic vascular functioning in diabetes mellitus in black patients.

## 1. Introduction

Diabetes mellitus (DM) is the most common metabolic disorder worldwide and affects an estimated 463 million people worldwide and 34.2 million people in the United States (U.S.)—about 10.5% of the U.S. population [1,2]. Furthermore, epidemiological studies demonstrate higher rates of DM in non-Hispanic black populations, as well as a higher risk of microvascular complications of DM including nephropathy progressing to end-stage renal disease (ESRD) and diabetic retinopathy (DR) compared to white populations in the U.S [3,4,5].

DR is the leading cause of blindness between the ages of 20–74 in the U.S. [6]. Changes in retinal microvascular structure associated with DR include pericyte and endothelial cell loss, decreased perfusion, and ultimately ischemia which leads to upregulation of pro-angiogenic factors (such as vascular endothelial growth factor [VEGF]) and subsequent neovascularization [7]. These changes are reflected throughout the rest of the body as well, which explains the strong association between DR and other microvascular complications of diabetes such as nephropathy and neuropathy [8]. In addition, recent studies have correlated microvascular complications in diabetics (including DR) with generalized vascular dysfunction and an increased risk of cardiovascular disease and all-cause mortality [9], independent of other risk factors such as diabetes duration, glycemic control, smoking, and lipid profile [10]. 

Optical coherence tomography angiography (OCTA) has added more precise noninvasive methods of analyzing retinal vasculature in recent years. OCTA allows researchers to analyze quantitative parameters such as superficial capillary plexus vessel density (SCP VD), deep capillary plexus vessel density (DCP VD), foveal avascular zone (FAZ) area, acircularity index (AI), and vascular length density (VLD) [11]. Using OCTA analysis, diabetics without DR have been shown to have decreased superficial and deep retinal VD in the parafoveal area, increased nonperfusion, and increased FAZ area compared to healthy subjects [7,12,13,14,15]. Moreover, evaluation of these changes using OCTA analysis can help predict an increased risk for progression of DR and development of diabetic macular edema (DME) [16], and worsening of these changes ultimately leads to poorer visual outcomes [14,17,18,19,20].

Differences between races on OCTA analysis have also been identified, as prior studies from our group have suggested that black populations may have decreased macular capillary vasculature at baseline compared to white populations even in the absence of systemic disease [21]. To date, limited data has been published regarding changes in retinal microvasculature in black subjects with diabetes and how these changes may correlate with systemic biomarkers. In this study, OCTA was used to analyze retinal vasculature and study correlations between retinal microvascular environment characteristics and systemic diseases in black adults with DM but without DR to help establish microvascular changes that correlate with systemic diseases in this population. These parameters could serve as a novel biomarker for microvascular complications especially in the black populations that are more vulnerable to diabetic microvascular complications.

## 2. Materials and Methods

This prospective, single-center, cross-sectional study of participants was approved by the Institutional Review Board of the University of Chicago (IRB #17-0170). All study protocols adhered to the tenets of the Declaration of Helsinki. The study conformed to the Health Insurance Portability and Accountability Act of 1996 regulations. The study was conducted between 1 February 2017 and 23 January 2019. All subjects provided informed, written consent.

### 2.1. Participants

Subjects were recruited at a single center, large, academic retinal practice at the University of Chicago Eye Clinic. These patients were established clinic patients that had been diagnosed with diabetes mellitus and had a recent dilated fundus exam and evaluation by a retina specialist that confirmed no evidence of DR. Inclusion criteria included age over 18 years, history of diabetes mellitus (type 1 or 2), and self-identification of black/African-American ethnicity. Exclusion criteria included diabetic retinopathy, medical conditions outside the variables listed in the study that would compromise microvasculature, and ocular conditions or ocular surgical history that would compromise image quality or retinal microvasculature. A history of systemic disease was treated as a binary variable (i.e., the subject either had or did not have the disease). Furthermore, many patients were currently being treated with pharmacologic agents (e.g., insulin, oral hypoglycemics, statins, antihypertensives, etc.). Subjects were prospectively consented for additional OCTA imaging using Optovue RTVue XR Avanti 2017.1 (3 × 3 mm macula cube) for further evaluation of retinal vasculature. All subjects were properly informed of the risks and benefits of additional imaging procedures, as well as the use of subsequent data gained from these imaging procedures. Chart review for demographic information, clinical information (history of other diagnoses including presence of hypertension, hyperlipidemia, heart disease [defined as past medical history including heart failure, coronary artery disease, and/or myocardial infarction], and smoking status), ocular history, and relevant laboratory values (creatinine, estimated glomerular filtration rate [GFR], albumin-to-creatinine ratio [ACR], total cholesterol, hemoglobin A1c [HbA1c]) of subjects was performed. Only images with a signal strength intensity (SSI) ≥0.5 were used in the analysis. 

### 2.2. OCTA Imaging

**Figure 1:**
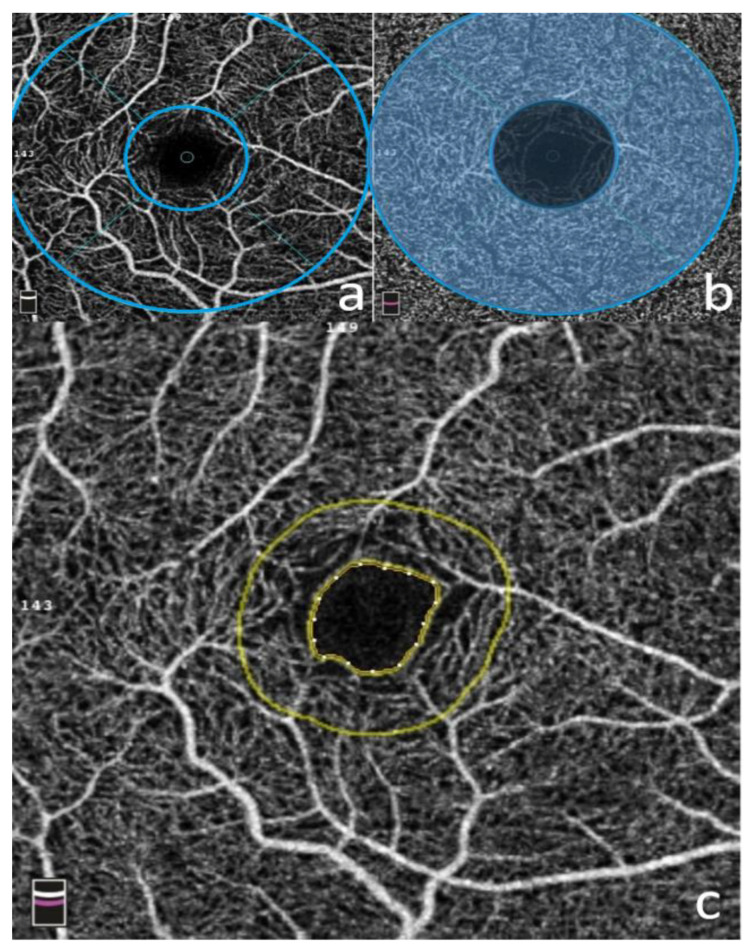
OCTA Software. Optovue RTVue XR Avanti 2017.1 (3 × 3 mm macula cube) software was used to analyze retinal microvasculature. Figure 1a represents superficial capillary plexus en-face image with 1mm and 3 mm rings surrounding fovea (blue). Figure 1b represents an image of the deep capillary plexus with 1–3 mm annulus representing the parafoveal region (transparent blue annulus). Figure 1c represents foveal avascular zone (FAZ) with marked perimeter (inner yellow circle) surrounded by 300 µm ring (outer yellow circle) used to calculate foveal vascular length density (FD300-LD) and foveal vascular area density (FD300-AD).

Images were obtained using the Optovue RTVue XR Avanti (Optovue Inc, Fremont, CA, USA Version 2017.1) with the AngioRetina mode (3 × 3 mm macular cube). Each image was made up of 304 clusters of repeated B-scans containing 304 A-scans, and the images were automatically segmented. AngioAnalytics software was used to analyze retinal vascular parameters after IRB approval. The software set the superficial capillary plexus (SCP) at the inner limiting membrane (ILM) and 9µm above the IPL (inner plexiform layer); the deep capillary plexus (DCP) was set between 9µm above the IPL and 9 µm below the outer plexiform layer (OPL).The superficial whole image was the entire 3 × 3 mm macular cube above the DCP. The parafoveal area was a 1–3 mm annulus surrounding the central fovea (see Figure 1). The FAZ (foveal avascular zone, in mm^2^) at the level of the SCP and DCP was manually measured with the built-in software. The acircularity index (AI) was defined as the ratio of the perimeter of the FAZ to the perimeter of a circle with equal area [22,23]. The foveal vascular length density (FD-300 LD) was determined as vessel density within a ring of a width of 300 μm surrounding the FAZ. The foveal vascular area density in the 300-μm ring (FD-300 AD) was calculated by dividing the number of vessel pixels by the total number of pixels then multiplied by 100%. The threshold for signal strength intensity (SSI) was set at ≥50 based on previous studies [24,25,26].

### 2.3. Vessel Density Analysis 

Vessel Density (VD) measurements were automatically generated by the software. It represents the percentage of pixels in the 3 × 3 mm macular scan that correspond to blood vessels that were automatically calculated. Within the 3 × 3 mm macular scan, there was an annulus of concentric circles of 1mm and 3mm diameters, with the inner circle defined as the fovea and the parafovea defined as the ring between the two circles. 

### 2.4. Statistical Analysis

Statistical analysis was performed using Stata13 (College Station, TX: StataCorp LP). To control for 2 eyes of the same patient, linear mixed models were used to obtain OCTA marginal means values ± standard deviation. Variables showing significant correlation with OCTA values on univariate mixed model analysis (*p* < 0.05) were analyzed with mixed-effects multivariate models, also controlling for 2 eyes of the same patient.

## 3. Results

92 eyes of 52 black adult subjects with DM were included in this study. 12 eyes were excluded from the study for meeting exclusion criteria for only one eye (signal strength intensity <0.5, ocular conditions that would affect microvasculature unilaterally but are unrelated to the study variables, and/or past ocular surgical history of the excluded eye). The mean age was 57.89 ± 15.85 (20.6–88.2 years, *p* = 0.1537). Most subjects were female (75%) and all the patients identified as black/African-American. Additional patient demographics and mean lab values are included in Table 1. 

The mean SSI was 65.06 ± 8.17. The mean superficial vessel density was 43.41 ± 5.13% in the whole image and 46.27 ± 5.39% in the parafovea. The mean deep vessel density was 48.71 ± 4.64% in the whole image and 51.23 ± 4.66% in the parafovea. The mean FAZ area was 0.34 ± 0.13 mm^2^. Additional measurements are included in Table 2. 

In the univariate mixed effect model for superficial whole image, age, SSI, heart disease, and Cr were statistically significant (age: −0.162, *p* < 0.001; SSI: 0.427, *p* < 0.001; heart disease: −3.536, *p* = 0.022; Cr: −5.198, *p* = 0.017) (Table 3a). The univariate analysis of the superficial parafovea had the same statistically significant variables (age: −0.15, *p* < 0.001, SSI: 0.45, *p* < 0.001, heart disease: −3.50, *p* = 0.028, Cr: −5.18, *p* = 0.018).

In the multivariate analyses for the superficial whole image VD and superficial parafovea, SSI and heart disease were statistically significantly correlated with VD. In the superficial parafovea, SSI had a positive association (0.40, *p* < 0.001), while having heart disease was negative (−2.82, *p* = 0.019) (Table 3b).

SSI and smoking status were significantly associated with the deep whole image VD (SSI: 0.182, *p* = 0.002, smoking status: −2.841, *p* = 0.011, respectively). In the multivariate analysis, only SSI remains statistically significant (0.154, *p* = 0.011). 

SSI and smoking status were significantly associated with deep parafoveal VD (0.14, *p* = 0.017; −2.6, *p* = 0.022, respectively). Through the multivariate model, none of the clinical factors studied were significant in the deep parafoveal VD.

FAZ area was significantly associated with Cr (−0.131, *p* = 0.015), and FAZ perimeter with pack-years of smoking in univariate analysis (0.018, *p* = 0.008). Both FAZ acircularity index and FD300-AD were associated with SSI in multivariate analysis (−0.005, *p* < 0.001; 0.491, *p* < 0.001, respectively). SSI and heart disease were shown to be statistically associated in the multivariate analysis for FD300-LD (SSI: 0.293, *p* < 0.001; heart disease: −1.943, *p* = 0.049). 

Hyperlipidemia, hypertension, smoking status, pack-years, DM duration, Cr, GFR, total cholesterol, HbA1C, and albumin-to-Cr ratio (ACR) were not significantly associated with any OCTA measurement in multivariate analysis.

## 4. Discussion

In this study, we analyzed the retinal vasculature of black subjects with DM without DR and analyzed correlations to systemic diseases. After multivariate analysis, SSI and heart disease had statistically significant correlations to SCP VD in our population. 

SSI and smoking status had statistical correlations to DCP VD in a univariate analysis that persisted in at least part of the imaging subset (e.g., deep whole image) in a multivariate analysis. None of the variables studied were associated with changes in deep parafoveal VD in a multivariate model of analysis.

In addition, none of the variables analyzed were statistically associated with FAZ area in a multivariate analysis, and SSI was the only variable that was significantly correlated with AI, FD300-AD, and FD300-LD.

Several of our results support previously published research, which is discussed below.

### 4.1. Age

Our study demonstrated a statistical correlation between age and decreased SCP VD on whole image and parafoveal imaging techniques in a univariate analysis, but these correlations were not shown to be statistically significant in a multivariate analysis. Additionally, only univariate analysis correlations were present between age and AI, FD300-AD, and FD300-LD, and none of these were statistically significant on multivariate analysis. 

Prior studies have shown that age is correlated with decreased retinal capillary density in healthy subjects [27,28,29,30], including black populations [31]. Additionally, healthy black subjects have shown to have an increased FAZ area at baseline compared to white subjects [21,32], and diabetic patients have also been shown to have an increased FAZ area compared to non-diabetic patients even in the absence of diabetic retinopathy [13,15]. Based on this prior data, we know that black populations as well as diabetic populations have baseline structural differences in retinal microvasculature compared to subjects of other ethnicities and non-diabetics. Our data suggests that these structural differences in black diabetic populations may make the retinal microvasculature less susceptible to age-related changes. However, while our study included subjects with ages of 20–88 years, most of our population were middle-aged, and we may not have had statistical power to detect true correlations between age and OCTA changes as we had only a few individuals that fell towards the extremes of this age range.

### 4.2. Signal Strength Intensity

Signal strength was shown to be positively correlated with VD in our study in many subsections of OCTA analysis, including superficial whole image VD, superficial whole VD, superficial parafoveal VD, deep whole image VD, FD300-AD and FD300-LD in a multivariate analysis. Furthermore, our study showed a weak negative correlation between SSI and AI. 

Yu et al. found on two OCTA platforms that VD measurements decreased linearly with decreasing signal strength with high statistical significance [30]. Similarly, Lim et al. found that VD, perfusion density (PD), and FAZ area significantly increased with increased signal strength [24]. In addition, Czakó et al. demonstrated that repeatability of OCTA metrics in diabetic populations are significantly affected by reduced signal strength [33]. 

The weak negative correlation with acircularity may have been related to processing of data, as vessels in images with less signal strength may have registered with the software as being more tortuous, and therefore deviating more from the circle of equal area that the software uses to calculate AI, due to projection artifacts associated with decreased signal strength. 

Similar to conclusions suggested by prior authors [24,30,33,34], our results highlight the importance of using high-quality images with a high level of signal strength when interpreting OCTA metrics, including black diabetic populations.

### 4.3. Heart Disease

In a multivariate analysis, our study demonstrated that heart disease was negatively correlated with SCP VD in whole image and parafoveal regions, and was the strongest correlation we found of the variables studied. This supports the results of prior research regarding OCTA metrics and associations with heart disease in other populations.

Multiple prior studies have correlated retinal vessel atherosclerosis and/or reduced vessel diameter with increased incidence of coronary artery disease and risk of death secondary to coronary events and stroke [35,36,37,38]. The methods of analysis used in these studies was direct visualization of retinal vasculature by examiners or analysis of fundus photographs. 

Limited data is available regarding the role of OCTA analysis in correlating quantitative changes in retinal microvasculature to heart disease; however, Wang et al. studied an Asian cohort of 316 subjects and compared coronary artery disease (CAD) patients to healthy controls. Their results showed that CAD patients had reduced retinal vessel density, choroidal vessel density, and flow area, and the authors concluded that OCTA may be a noninvasive strategy for identifying high-risk early stage CAD individuals that may benefit from further examination or cardiac procedures. Furthermore, they proposed that the mechanisms responsible for these microvascular changes are likely similar to those seen in larger vessels contributing to CAD, including atherosclerotic changes to the vessel walls that result in thickening and stenosis. Their study found that vessel density was directly correlated with Gensini score, which is a well-established weighted grading system of coronary artery stenosis that grades based on which coronary arteries are stenotic (left main coronary artery [LMCA] carrying the most weight, followed by left anterior descending branch [LAD], left circumflex coronary artery [LCX], etc.), and found that SCP VD and DCP VD, as well as choroidal capillary vessel density, was directly correlated to Gensini score. In other words, a greater degree of retinal and choroidal vessel density loss was present in those patients which had LMCA stenosis, followed by LAD and LCX stenosis, which correlates well to severity of coronary artery disease [39]. 

Our results support the results of Wang et al., and suggest that similar findings, at least in the superficial retinal vasculature layer, are found in not only an Asian population, but also the black diabetic population of our study. This provides further support that OCTA parameters may be an under-utilized tool in screening patients for early stage CAD that may warrant further evaluation. 

### 4.4. Creatinine (Cr), Estimated Glomerular filtration Rate (eGFR), and Albumin-to-Creatinine Ratio

Creatinine was negatively associated with SCP VD, FAZ area, and FD300-AD, FD300-LD, and weakly positively associated with increased AI in a univariate analysis, but none of these associations were found to persist in a multivariate analysis. eGFR was positively associated with FD300-AD and FD300-LD in a univariate analysis but not in a multivariate analysis.

Some prior studies of Chinese populations have shown that higher creatinine level is associated with decreased retinal vessel density [34] and lower eGFR is associated with increased FAZ size in diabetic populations [15], while other studies with similar populations failed to show any similar correlations [14,40]. While microvascular complications of diabetes such as nephropathy are more common in black diabetic populations [3,4,5], our population likely included diabetics with relatively mild microvascular complications as demonstrated by their lack of retinopathy on exam and low average creatinine. Further studies of OCTA parameters including black diabetic patients with DR and more severe kidney dysfunction may better elucidate further correlations. 

### 4.5. Smoking Status and Smoking Pack-Years

Current smoking status was negatively correlated with DCP VD in the whole image and parafoveal image analysis in our study in a univariate analysis but failed to show a correlation in a multivariate analysis. Additionally, our results showed a positive correlation with AI, and a negative correlation in FD300-AD and FD300-LD in a univariate analysis. Additionally, pack-years smoking was positively correlated with FAZ perimeter in a univariate analysis. However, none of these changes were statistically significant on multivariate analysis. 

Lee et al. found that current smoking status was associated with decreased DCP VD but not SCP VD reduction in a diabetic population [15]. These results align with other studies that have shown decreased blood flow [41] and reduced retinal capillary density in smoking diabetic populations [42]. Our study population had a relatively low number of former or current smokers (25/52), which may be the reason that smoking status was not correlated to OCTA parameters analysis and we may not have had adequate statistical power to detect these correlations in the multivariate analysis and was not identified in the univariate.

### 4.6. HbA1C and Diabetes Duration

Our study did not show any statistical associations in OCTA parameters with HbA1c level and diabetes duration in either univariate or multivariate analysis. Prior studies in Chinese and African American diabetic populations also failed to show correlations with HbA1c (26, 36), although diabetes duration was correlated with reduced capillary vessel density on OCTA in a previous study of diabetic African-American subjects [31]. Our results, at least in part, highlight the multi-factorial nature of diabetes. While previous studies have shown that the risk of diabetic retinopathy increases with higher HbA1c levels and longer diabetes duration, especially in racial/ethnic minorities [3], our data may suggest that those eyes that do not exhibit clinical signs of disease also do not seem to have sub-clinical OCTA parameter changes correlated to glycemic control. It is possible that some eyes respond more robustly to changes in glycemic control (and therefore develop clinical retinopathy), while eyes such as the ones that were used in our study are less affected and may be more tolerant to these changes, even at the level of detail detectable by OCTA analysis. More data is needed to explain the mechanisms responsible for the differing response severity in these patients.

### 4.7. Other Variables (Hypertension, Hyperlipidemia, Total Cholesterol)

Our results did not show any correlations between OCTA parameters and hypertension, hyperlipidemia, or total cholesterol in either univariate or multivariate analysis. Limited previously published data is available for these variables with similar characteristics to our patient population, which makes these results difficult to compare to prior research. Changes in microvascular structure of the choriocapillaris on OCTA has been associated with hypertension in a Japanese study population [43], and hypertension has been shown to be associated with lower VD in diabetic patients [15] as well as otherwise healthy individuals [44] in Korean populations. Furthermore, lower VD has been associated with dyslipidemia in populations in South Korea [15] and Singapore [42]. These results highlight the need for analyzing OCTA parameters in diverse patient populations, as these changes may be due to ethnic differences among these populations or may simply be due to inter-study variability.

### 4.8. Limitations

Firstly, our population was a relatively small and homogenous population at a single-center academic retinal practice in Chicago, IL. The subject recruitment in the study was limited to available subjects that met inclusion and exclusion criteria in our single-center practice. Due to lack of normative OCTA vascular data for this patient population, upon which we could draw an estimated effect size, formal power calculations were not possible for this study. However, we believe our data serves as a starting point for future studies that investigate the establishment of normative OCTA databases in this population, and we acknowledge that we cannot claim with certainty that the differences found in this study would be applicable to a broader population. In the future, a larger study could potentially identify additional factors associated with OCTA parameters. 

We decided to narrow our population to black diabetic subjects as the black population remains underrepresented in scientific literature [45] despite microvascular complications of diabetes being more prevalent in this population [3,4,5]. Previous studies have shown that young black patients without systemic disease have differences in OCTA parameters [21], which is why we focused on black patients with diabetes. Studying patients without retinopathy prevented retinal hemorrhage, cysts, or exudates from influencing OCTA parameters. This pilot study can help aid in the design for future studies in this patient population and ensure less bias is introduced in the interpretation of OCTA parameters and association in retinal disease burden. That being said, a larger data set from more subjects over a larger and more diverse geographical area would help add statistical power to the results found in this study. Similarly, future studies including both comparison groups of additional ethnicities and black patients without diabetes would further aid in establishing the effect of race/ethnicity in OCTA measurements. 

Secondly, a potential confounder of any study comparing OCTA parameters to other previously published research is that OCTA analysis methods have been shown to lack interchangeability with one another [46]. This makes interpretation of data difficult, as inter-study variability exists in methods used for imaging and post-imaging processing and analysis. To an extent, this unfortunately also limits the uses of normative databases until a standard method of OCTA analysis is widely adopted.

Thirdly, a confounder that is inherent to studies using OCTA is the role of axial length in OCTA parameter analysis. Previous studies have shown that axial length has been shown to affect vessel density and FAZ area [14,28], but unfortunately not widely adopted and verified magnification correction factor has been developed to date, so this variable is difficult to correct. Unfortunately, axial length data was not available for analysis for our subset of patients, so conclusions drawn from our data could potentially be skewed by a non-gaussian distribution of axial lengths among our population. 

Lastly, as described above, systemic diseases other than diabetes were treated as binary variables upon chart review, and we did not further stratify based on disease duration, severity of disease, and use of pharmacologic treatment agents. Hypertension, for example, has varying degrees of severity, and long-standing uncontrolled severe hypertension is likely much more damaging to microvasculature than milder cases of disease. Ultimately, more data is needed to further stratify whether these correlations persist if these diseases are diagnosed early and well-controlled.

## 5. Conclusions

Our findings suggest that OCTA measures may serve as valuable biomarkers in black patients to track systemic vascular functioning in DM and underscore the importance of working towards establishing normative databases that represent diverse populations. Clinically, these findings suggest OCTA may be helpful in identifying microvascular characteristics that may guide the physician to refer these patients for closer monitoring of other systemic diseases, such as heart disease. Furthermore, our data reflects that the presence of systemic diseases have correlations with baseline OCTA parameters in black diabetic populations. This highlights that baseline ethnic characteristics of study participants as well as the presence of systemic diseases need to be considered when analyzing OCTA imaging, especially when being used for research purposes. Further studies including larger sample size of patients with systemic diseases from diverse racial backgrounds are needed to further delineate the correlations of systemic vascular status with retinal microvascular environment and their involvement in retinal microvascular diseases.

## Figures and Tables

**Table 1:** Patient Demographics and Clinical Characteristics.

	Subjects (*n* = 52)
Baseline criteria	
Female gender, *n* (%)	39 (75)
DM, years, mean (SD)	8.79 (7.29)
Former or current smoker, *n* (%)	25 (48)
Pack-years, mean (SD)	6.11 (9.16)
Hyperlipidemia, *n* (%)	18 (35)
Hypertension, *n* (%)	31 (60)
Heart Disease, *n* (%)	11 (21)
Lab Values	
Creatinine [Cr] (SD)	0.89 (0.31)
Glomerular Filtration Rate [GFR] (SD)	78.15 (19.63)
Albumin-to-Cr Ratio [ACR] (SD)	186.33 (460.49)
Total Cholesterol (SD)	197.71 (157.06)
HbA1c (SD)	7.64 (1.98)

**Table 2:** Vessel Density in Superficial and Deep Vascular Capillary Plexus and FAZ measurements.

	Vessel Density (*n* = 92 Eyes)
SSI	65.06 ± 8.17
Superficial vessel density (%)	
Whole image	43.41 ± 5.13
Parafovea	46.27 ± 5.39
Deep vessel density (%)	
Whole image	48.71 ± 4.64
Parafovea	51.23 ± 4.66
FAZ	
FAZ area (mm^2^)	0.34 ± 0.13
Perimeter (mm)	2.35 ± 0.48
Acircularity index [AI]	1.16 ± 0.08
FD-300 area density (%)	48.41 ± 7.05
FD-300 length density (%)	15.38 ± 3.82

**Table 3:** (**a**) Results of Univariate Analysis. Correlation coefficients are provided for all parameters and variables. Values that are bolded are statistically significant (*p* < 0.05) and the 95% confidence interval is provided. (**b**) Results of Multivariate Analysis. Correlation coefficients are provided for all parameters and variables. Values that are bolded are statistically significant (*p* < 0.05) and the 95% confidence interval is provided. Variables and parameters that did not have multiple statistically significant variables in the univariate analysis were not included in this table.

**(a)**									
**Variables**	**Superficial Whole Image**	**Superficial Parafovea**	**Deep Whole Image**	**Deep Parafovea**	**FAZ Area**	**Perimeter**	**Acircularity Index**	**FD300 Area Density**	**FD300 Length Density**
Age	−0.162 (−0.238 to −0.086)	−0.153 (−0.23 to −0.07)	−0.073	−0.06	0.001	0.005	0.001 (0.000 to 0.002)	−0.213 (−0.311 to −0.116)	−0.146 (−0.199 to −0.092)
SSI	0.427 (0.332 to 0.523)	0.454 (0.350 to 0.560)	0.182 (0.068 to 0.297)	0.142 (0.030 to 0.260)	0.001	0.000	−0.003 (−0.005 to −0.002)	0.521 (0.376 to 0.666)	0.342 (0.273 to 0.410)
Hyperlipidemia	0.260	0.46	1.869	2.12	0.013	0.040	−0.014	1.297	0.326
Hypertension	1.017	1.33	−1.715	−1.72	0.022	0.068	−0.011	1.301	0.343
Heart disease	−3.536 (−6.563 to −0.509)	−3.498 (−6.563 to −0.509)	−1.392	−0.71	0.045	0.197	0.014	−4.365 (−8.350 to −0.379)	−3.264 (−5.514 to −1.015)
Current smoker	−1.859	−1.72	−2.841 (−5.021 to −0.660)	−2.600 (−4.830 to −0.370)	0.038	0.222	0.053 (−0.005 to −0.002)	−3.321(−6.641 to −0.002)	−2.500 (−4.409 to −0.591)
Pack years	−0.069	−0.07	−0.087	−0.06	0.004	0.018 (0.005 to 0.032)	0.003 (0.000 to 0.005)	−0.047	−0.071
DM duration	−0.057	−0.06	0.005	0.02	0.004	0.013	0.000	0.036	−0.015
Cr	−5.198 (−9.459 to −0.937)	−5.184 (−9.480 to −0.890)	−3.597	−3.76	−0.131 (−0.238 to −0.025)	−0.362	0.058 (0.001 to 0.115)	−6.371 (−12.178 to −0.565)	−3.750 (−7.255 to −0.244)
GFR	0.072 (0.004 to 0.140)	0.07	0.051	0.05	0.001	0.003	−0.001	0.101 (0.007 to 0.195)	0.064 (0.008 to 0.120)
Total cholesterol	−0.001	0.00	0.004	0.00	0.000	−0.001	0.001	−0.001	0.001
HbA1C	−0.142	−0.18	0.299	0.40	0.013	0.030	−0.007	0.716	0.342
ACR	0.002	0.00	0.002	0.00	0.000	0.000	0.000	0.001	0.002
**(b)**									
**Variables**	**Superficial Whole Image**	**Superficial Parafovea**	**Deep Whole Image**	**Deep Parafovea**			**Acircularity Index**	**FD300 Area Density**	**FD300 Length Density**
Age	−0.001	0.052					−0.001	−0.016	−0.007
SSI	0.403 (0.278 to 0.527)	0.475 (0.350 to 0.60)	0.154 (0.036 to 0.272)	0.115			−0.005 (0.007 to −0.002)	0.491 (0.274 to 0.708)	0.293 (0.197 to 0.389)
Heart disease	−2.819 (−5.531 to −0.424)	−2.795(−5.200 to −0.390)						−3.176	−1.944 (−4.561 to −0.0535)
Current smoker			−2.118	−2.053			−0.059	−0.503	−0.823
Pack years							0.001		
Cr	−4.459	−2.577					0.012	−3.382	−0.472
GFR	−0.039							−0.025	−0.001

## Data Availability

The data presented in this study are available on request from the corresponding author.
